# Thiol-based chemically modified carbon screen-printed electrode for simultaneous quantification of trace level Pb(II) and Cd(II)

**DOI:** 10.1007/s44211-024-00581-z

**Published:** 2024-05-14

**Authors:** Mritunjay S. Tiwari, Arun K. Kadu

**Affiliations:** University Department of Chemistry, University of Mumbai, Vidyanagari, Santacruz (East), Mumbai, 400 098 India

**Keywords:** Electrochemical sensor, SWASV, Cysteamine, Screen-printed carbon electrode, Heavy-metal ions

## Abstract

Present work reports, the development of a novel electrochemical sensor based on a diazonium-coupling reaction and covalent attachment of the –NH_2_ group of cysteamine (Cyst) on screen-printed carbon electrode (SPCE), for simultaneous determination of Pb(II) and Cd(II). Initially, the in-situ generated 4-carboxyphenyl (4-CP) diazonium salt was electro-grafted to generate 4-CP/SPCE, followed by covalent bonding of terminal carboxylic group of 4-CP with –NH_2_ group of Cyst to give Cyst/4-CP/SPCE. The modified electrode showed an enhanced selectivity and sensitivity toward the quantification of Pb(II) and Cd(II) using square wave anodic stripping voltammetry (SWASV) without mutual interference. Under optimal experimental conditions, the newly designed sensor showed a wide linear range of 0.01 µM to 0.7 µM. The limit of detection (LOD) obtained was 0.882 nM (0.09 ppb) and 0.65 nM (0.134 ppb) for Cd(II) and Pb(II), respectively. The modified SPCE exhibited good stability, selectivity, and reproducibility. Furthermore, the sensor was successfully applied for the determination of Pb(II) and Cd(II) ions in water samples which illustrated excellent recoveries in different spiked samples and the results were in line with the standard ICP-AES analysis.

**Graphical abstract:**

A thiol-based disposable electrochemical sensor was developed via electro grafting of diazonium salt on SPCE followed by covalent immobilization of cysteamine for quantification of Pb(II) and Cd(II) in water samples.

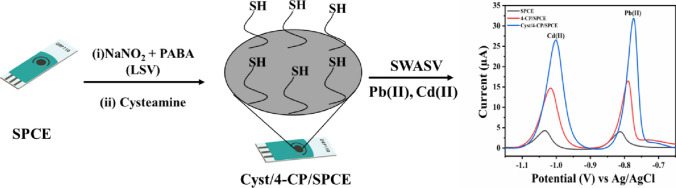

**Supplementary Information:**

The online version contains supplementary material available at 10.1007/s44211-024-00581-z.

## Introduction

Heavy-metal ions such as Pb(II), Cd(II), As(III), and Hg(II), cause environmental pollution and contamination of groundwater. Due to nonbiodegradability and perseverance, these toxic metal ions accumulate in the environment and through the food enter in living organisms, causing harmful effects on human health [1, 2]. Cadmium and lead ions are two major metal ions widely found in wastewater and groundwater. A large quantity of inorganic lead comes from mining, various industrial fuels, and leaded petrol which eventually enters into natural water systems [3, 4]. Some surface and subsurface waters contain cadmium resulting from welding, electroplating, nickel–cadmium batteries, nuclear fission reactors, paints and plastics, fertilizers, and various other sources [5, 6]. The maximum contamination level (MCL) of Pb(II) and Cd(II) in drinking water has been set at an extremely low concentration of 50 ppb and 5 ppb, respectively [7, 8]. Accumulation of these metal ions has shown negative impacts on neurologic development in children, hypertension, reproductive system, and nephrological damage in humans [9, 10]. Therefore, monitoring and detecting metal ions at trace levels is necessary for water-quality control [11].

Currently, the monitoring and quantification of heavy-metal ions are done by one of the following conventional techniques such as atomic absorption spectrometry (AAS) [12], atomic fluorescence spectrometry (AFS) [13], inductively coupled plasma-mass spectrometry (ICP-MS) [14], and inductively coupled plasma-atomic emission spectrometry (ICP-AES) [15]. However, conventional techniques have low throughput, portability, and high-cost limitations because of their complicated instrumentation and laborious analysis [16]. Moreover, the majority of the aforementioned techniques are capable of analyzing a single metal ion at a time [11].

To quantify toxic metal ions like Pb(II), Cd(II), As(III), Hg(II) etc., electrochemical techniques particularly anodic stripping voltammetry (ASV) have been used. The electrochemical sensor has numerous advantages, such as enhanced selectivity, exceptional sensitivity, and portability along with low cost of analysis [17, 18]. Various studies reveal, SWASV as a simple, efficient, and sensitive electro-analytical technique, for trace-level determination of heavy-metal ions resulting from different clinical, and industrial activities [19]. In recent reports, use of the SWASV technique with various modified electrodes has been studied for trace-level analysis of Pb(II) and Cd(II) [20–22].

The use of screen-printed electrodes (SPE) as electrochemical sensors for the determination of heavy-metal ions has gained immense attention as they are cheaper and are suitable for on-site analysis. The selectivity and sensitivity of SPE toward metal ion sensing have been significantly improved due to the advancement in modifying materials used for sensors [23, 24]. One significant component to increase the sensitivity of SPE toward heavy-metal ions from the perspective of chemical interaction is electrode modification with functional groups. Oxygen-containing functional groups like carboxylic acid, hydroxy, and carbonyl, nitrogen-containing functional groups like nitrile, primary and secondary amines, and sulfur-containing functional groups like sulphoxide and thiols are some examples. Due to the high affinity of hetero atoms toward metal ions, they help in the pre-concentration process by selective binding of target metal ions on the electrode surface [25, 26].

Hwang et al. [27], modified planar carbon electrodes for the detection of Pb(II) and Zn(II) using chitosan (Cts). The Cts layer on the electrode has been found to facilitate the formation of metal chelates with analyte through its hydroxyl and amino functional groups. The modified electrodes were able to achieve a LOD of 1 ppb for Pb(II) and 0.16 ppb for Zn(II). Manikandan et al. [28] developed an electrochemical sensor for the determination of Hg(II) by DPV in fish muscle based on the anodized SPE (ASPE) modified by electrochemical polymerization of 5-amino-4H1,2,4-triazole-3-thiol (ATT) to form a poly-ATT (PATT) film. The developed sensor exhibited enhanced selectivity, sensitivity, and a low-detection limit of 0.005 nM. In other work, Li et al.[29] used thiol functionalized N-doped multi-walled carbon nanotubes (MWCNTs) modified glassy carbon electrode (GCE) for determination of Pb(II) and Cd(II) by SWASV in lab samples. The results indicated that due to the incorporation of thiol groups, N-doped MWCNTs acquired improved selectivity for metal ions detection. The modified electrode was able to detect Cd(II) and Pb(II) at a wide range of concentrations and provided a LOD of 0.4 µg L^−1^ and 0.3 µg L^−1^, respectively.

In a recent work, Yang et al. [30] studied the influence of cysteamine-functionalized graphene (GSH) as an electrode material for increasing the sensitivity of screen-printed carbon electrode (SPCE) toward the detection of Cd(II), Pb(II), and Cu(II) using SWASV. According to Pearson’s hard and soft acid and base concept, the grafted thiols on GSH sheets with excellent affinity toward heavy-metal ions could be responsible for the increase in sensitivity. The developed sensor exhibited a wide linear range of detection up to 200 ppb and a lower limit of detection for Cd(II), Pb(II) and Cu(II) were 15 ppb, 11 ppb, and 6 ppb, respectively.

Hence, the previous reports suggest that the thiol-modified materials or polymers containing the -SH functional group can be successfully used to prepare electrochemical sensors for the trace-level detection of heavy-metal ions. Thus, considering the chelating property of the -SH group with heavy-metal ions, we have developed an electrochemical sensor based on chemically modified SPCE using a diazotization coupling mechanism followed by covalent immobilization of cysteamine (Cyst).

## Experimental

### Chemicals and reagents

Sodium nitrite (99%), 99% p-amino benzoic acid (PABA), cysteamine hydrochloride (96%), potassium ferricyanide (98.5%), potassium ferrocyanide (99%), and potassium chloride (99%) was obtained from Sigma- Aldrich. Lead nitrate (99%), cadmium chloride (99%), sodium acetate trihydrate (99%), glacial acetic acid (99.5%), and all other reagents were provided by SD Fine Chemicals. SPCE (DRP-110) was procured from Metrohm, USA. All of these chemicals were of A.R. grade and used as received. Ultrapure deionized water (DI) was used to prepare all the solutions.

### Instrumentation

Electrochemical studies were performed on Autolab PGSTAT204 equipped with NOVA software (version 2.1.1). SPCE, with Ag/AgCl reference electrode, carbon as counter, and working electrode, was used for modification and analysis in this study. An ELICO LI 120 pH meter was used for all pH measurements. ICP-AES was carried out for real spiked samples on ARCOS, Simultaneous ICP Spectrometer.

### Surface modification of electrode

**Fig. 1:**
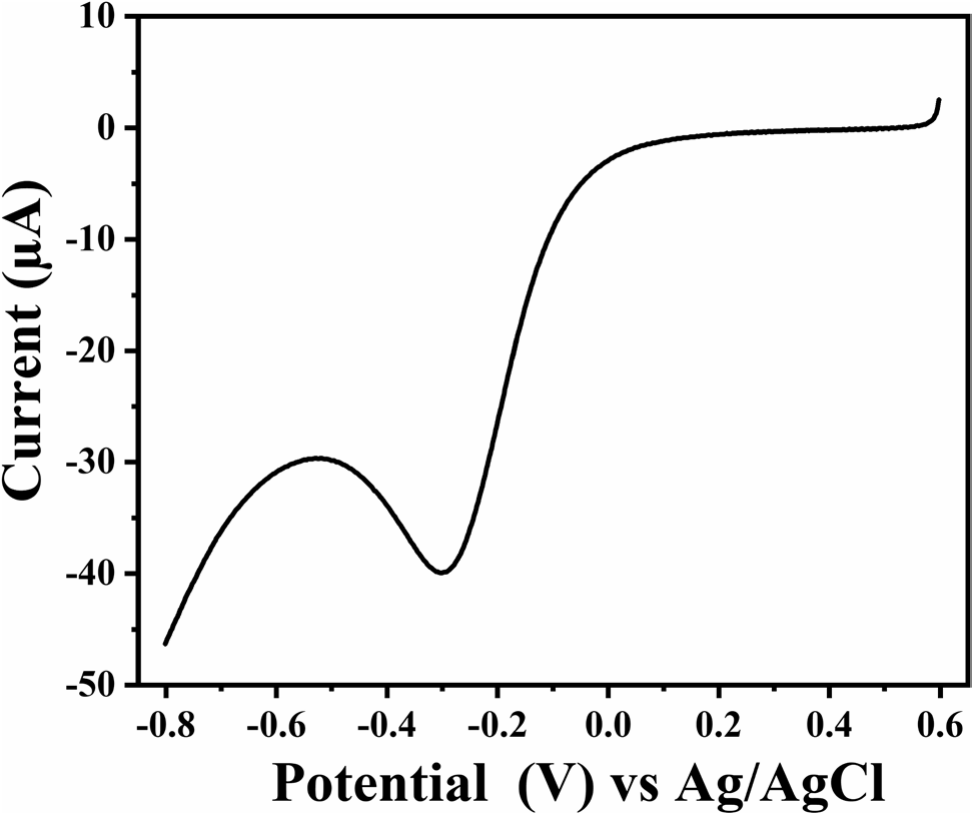
LSV plot for electro-grafting of 4-CP diazonium salt using diazotization mixture (1:1) NaNO_2_ + 4-aminobenzoic acid in 0.5 M HCl at SPCE

Prior to modification, SPCE was rinsed with 70% ethanol followed by DI and it was subjected to electrochemical pretreatment by CV between 1.0 V and − 1.5 V for 5 cycles in 0.5 M H_2_SO_4_. Surface modification of the electrode was carried out via. in-situ generated diazonium cations and was performed in three steps [31]. First, 4 mM of sodium nitrite was added in equal volume to the solution containing 4 mM of PABA prepared in 0.5 M HCl under constant stirring for 5 min under cold conditions. Second, the electro-grafting of in-situ generated 4-CP diazonium salt was done by linear sweep voltammetry (LSV) by scanning the potential from 0.6 V to − 0.8 V at a scan rate of 50 mV s^−1^ [32]. The modified electrode 4-CP/SPCE was rinsed with DI and dried under a stream of N_2_ gas. Third, the -COOH group on the electrode surface was condensed with the –NH_2_ group of Cyst by adding 20 µL of 2 mM cysteamine hydrochloride (Cyst) in 0.1 M phosphate buffer (pH 7) over the working electrode of 4-CP/SPCE for 3 h at room temperature. After immobilization, the electrode was rinsed with DI and the final modified electrode labeled as Cyst/4-CP/SPCE was used for further studies (Fig. 1).

### Real-sample preparation

Water aliquots from three different sources (drinking, tap, and wastewater) were collected and filtered through a 0.2 µ PVDF membrane syringe filter for real sample analysis. Drinking and tap water was collected from the University of Mumbai, Kalina Campus, Mumbai, and the wastewater was sampled from the Wagle estate industrial area, Thane, Maharashtra. 5 mL of each sample was collected in centrifuge tubes and the pH of the solution was adjusted to pH 4.5 using 0.1 M sodium acetate buffer. The recovery study was carried out using the standard addition method.

### Electrochemical detection of Cd (II) and Pb (II)

The lead and cadmium ion determination using Cyst/4-CP/SPCE was performed by SWASV in 0.1 M acetate buffer solutions (pH = 4.5). The frequency, step, and pulse amplitude for SWASV were 25 Hz, 1 mV, and 20 mV, respectively. The potential of − 1.1 V was applied for 240 s during the pre-concentration process under stirring conditions and finally, after equilibration, SWASV was performed between − 1.2 V to − 0.6 V.

## Results and discussion

### Functional group characterization of modified electrodes

**Fig. 2:**
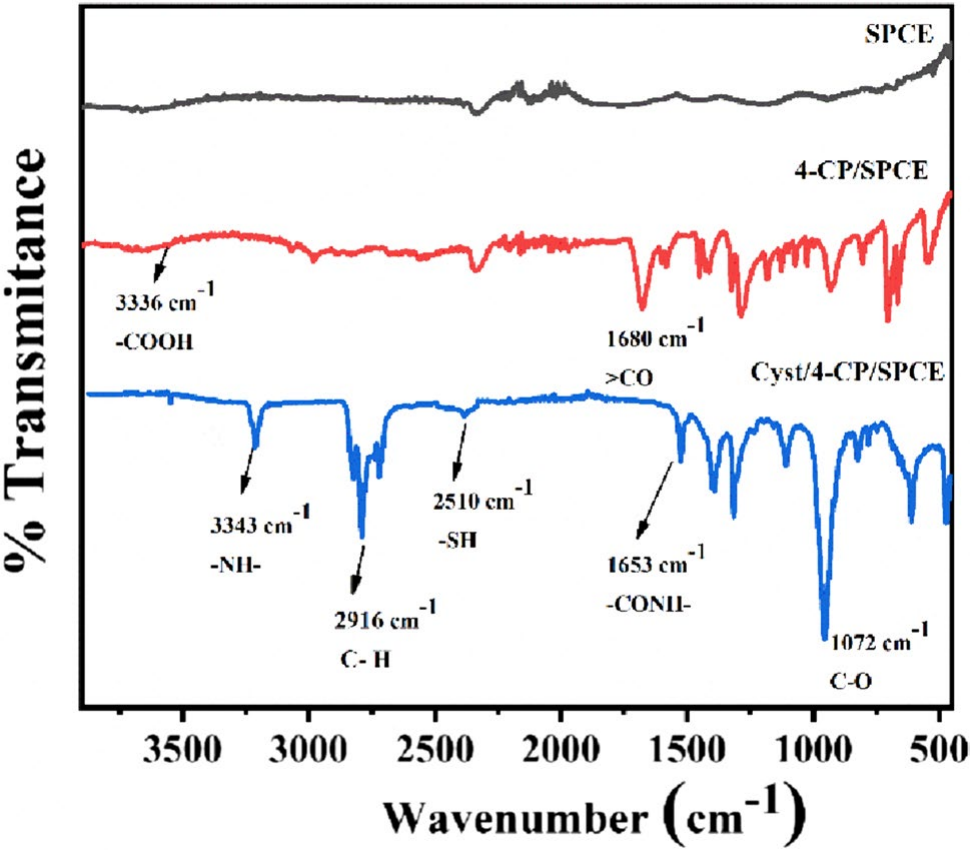
FTIR spectrum for SPCE, 4-CP/SPCE, and Cyst/4-CP/SPCE

FTIR spectrum of the bare and modified electrodes were recorded within the frequency range of 400 cm^−1^ to 4000 cm^−1^ [Fig. 2]. 4-CP modified SPCE shows a characteristic > CO peak at 1680 cm^−1^ and a broad peak at 3336 cm^−1^ for -COOH functional group confirms electro-grafting of 4-CP diazonium cation on SPCE. The attachment of the cysteamine to the terminal carboxylic group of 4-CP/SPCE is evident from the peaks observed at 3343 cm^−1^ and 1653 cm^−1^ corresponding to the -NH- and conjugated > C = O groups of amide, respectively. A typical weak peak at 2510 cm^−1^ confirms the presence of -SH group.

### Electrochemical characterization

**Fig. 3:**
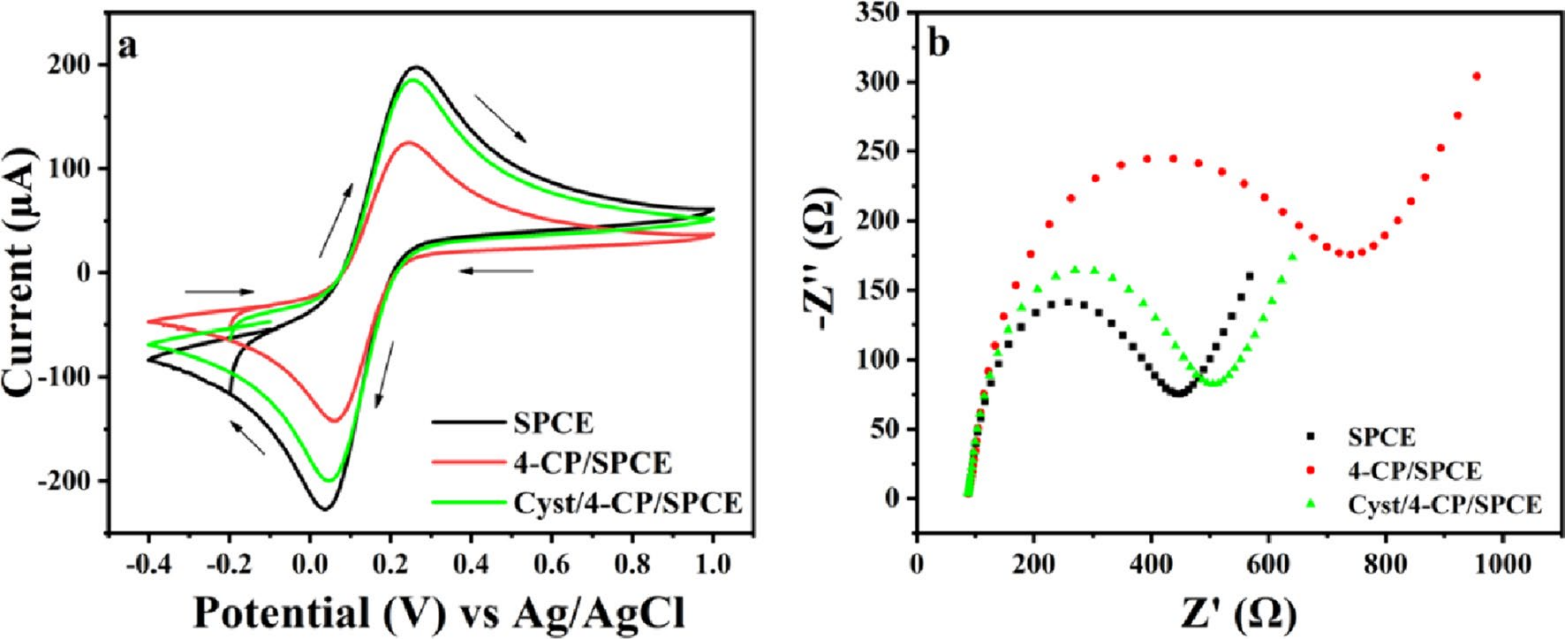
**a** CV plots of different modified electrodes **b** Nyquist plot of different modified electrodes using 5 mM of [Fe(CN)_6_]^3−/4−^ in 0.5 M KCl

The modified electrodes were electrochemically characterized by a solution of 0.5 M KCl containing 5 mM of [Fe(CN)_6_]^3−/4−^ as redox probe at a scan rate of 100 mV s^−1^. Figure 3a shows CV curves of bare SPCE, 4-CP/SPCE, and Cyst/4-CP/SPCE. The redox peaks obtained for 4-CP/SPCE were less than bare SPCE, which may be attributed to the covalent attachment of 4-CP with the electrode surface. An increase in peak current was observed after cysteamine immobilization onto 4-CP modified electrode. Variation in peak current after modification is seen because surface modifications may alter the active sites on the electrode surface. Changes in electroactive surface area due to modification can affect the available surface area for electrochemical reactions, thereby impacting the observed peak current.

Electrochemical impedance spectroscopy (EIS) also provides information on the barrier properties of the modified electrode. Figure 3b depicts the Nyquist plots for different modified electrodes. The results obtained were very similar to the CV shown in Fig. 3a

### Electrochemical behavior of Cd(II) and Pb(II)

**Fig. 4:**
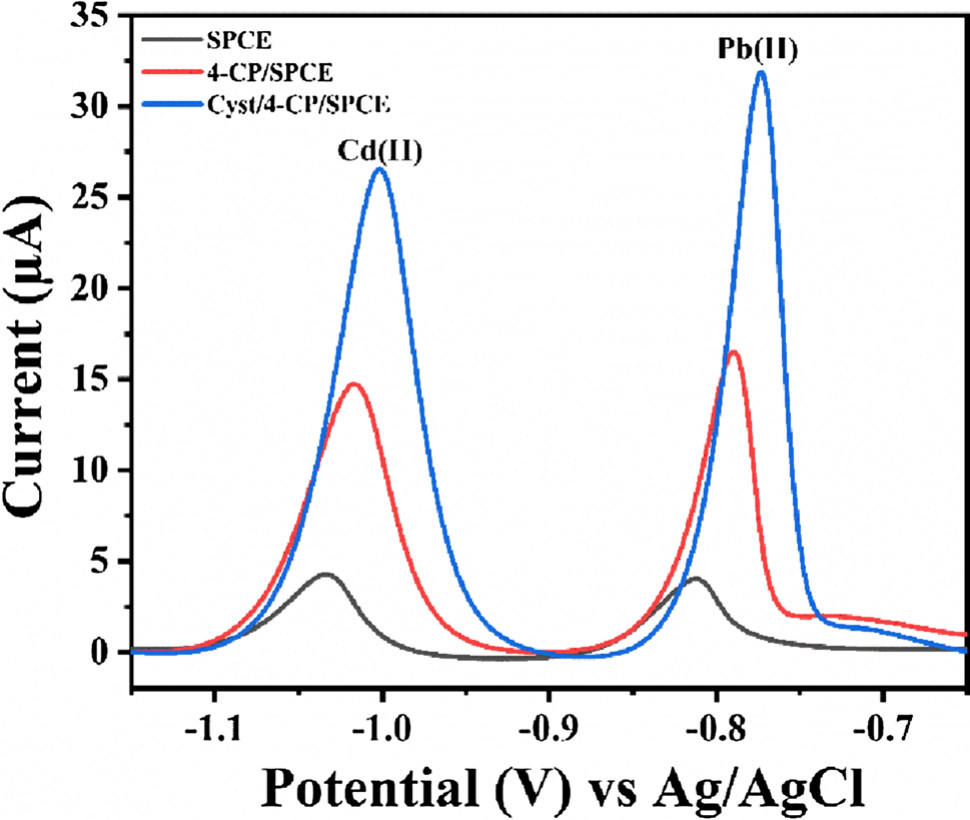
Square wave anodic stripping voltammograms for 0.5 µM of Cd(II) and Pb(II) ions in acetate buffer pH 4.5 on SPCE, 4-CP/SPCE, and Cyst/4-CP/SPCE

**Scheme 1.**
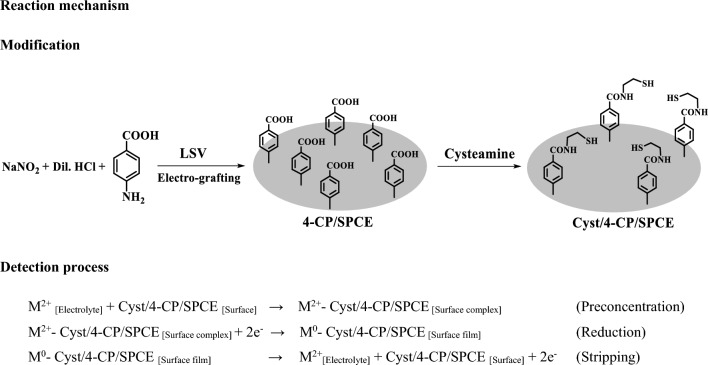
Representation of immobilization of cysteamine at SPCE, and the working principle of sensor based on metal–ligand complex formation

Figure 4 represents SWASV peaks at stepwise modified electrodes for 0.5 µM of Cd(II) and Pb(II) ions in acetate buffer pH 4.5, at a deposition potential of − 1.1 V with a pre-concentration time of 120 s. It can be seen from the figure that the reduction peak current obtained at Cyst/4-CP/SPCE for both the ions is greater as compared to 4-CP/SPCE modified electrode and bare SPCE. This enhanced activity of Cyst/4-CP/SPCE for the determination of cadmium and lead ions is due to the presence of the -SH functional group present at the terminal of conjugated cysteamine. As well known, heavy-metal ions (soft acids) can be immobilized on the electrode surface via thiol linkages (soft base), because of the high chelating ability of -SH group [33, 34] (Scheme 1).

## Reaction mechanism

### Modification

### pH and deposition time study

**Fig. 5:**
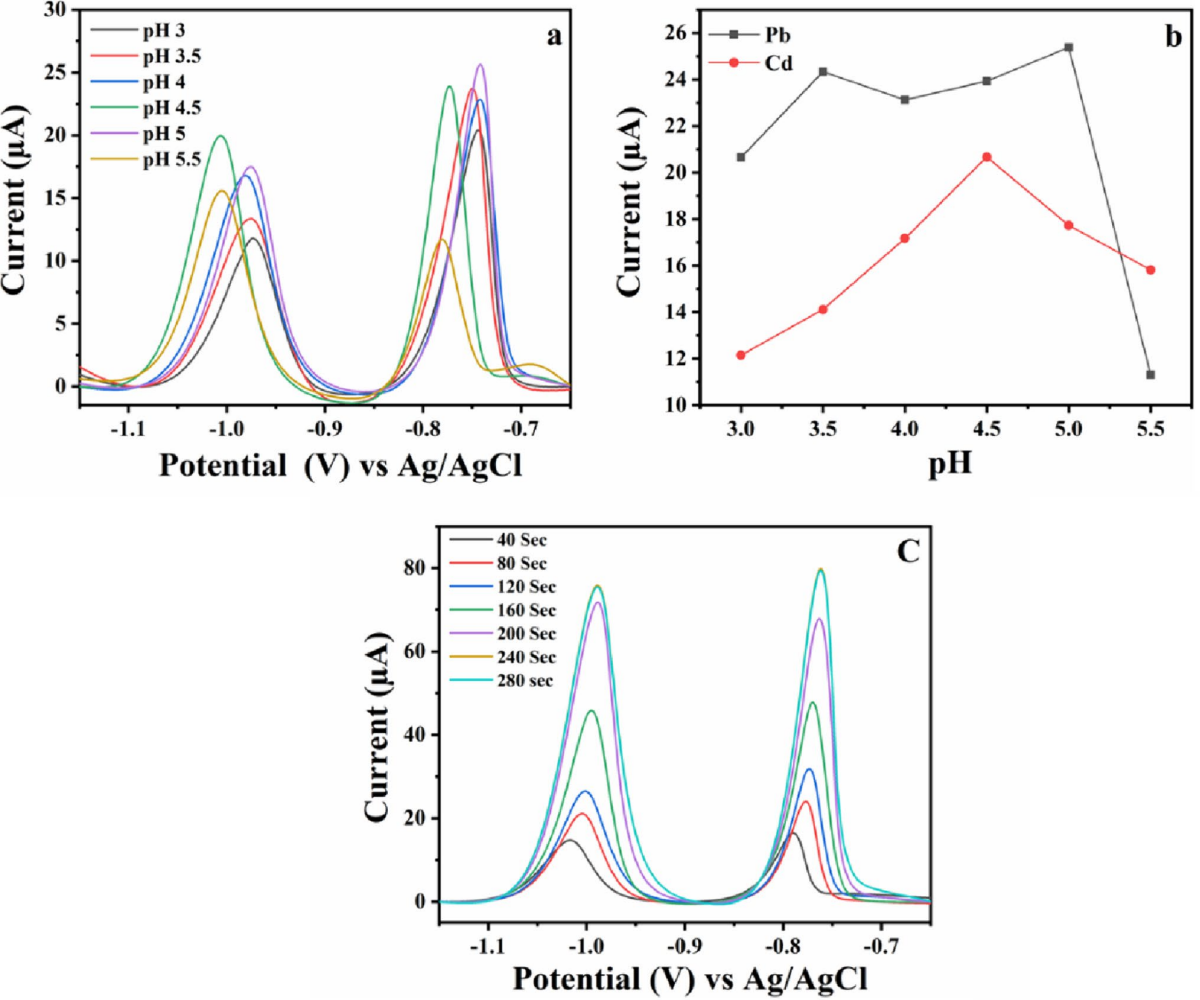
**a** SWASV, **b** pH vs peak current at Cyst/4-CP/SPCE for 0.5 µM of Pb(II) and Cd(II) in 0.1 M sodium acetate buffer at different pH (3 to 5.5), **c** SWASV plots with increasing deposition time for 0.5 µM of Pb(II) and Cd(II) in 0.1 M sodium acetate buffer pH 4.5

Optimization of pH was done by SWASV with a deposition potential of − 1.1 V and the deposition time was kept at 120 s. Sodium acetate and acetic acid were preferred to prepare buffer solutions of different pH over sodium citrate and Britton– Robinson buffer due to its stability, ease of preparation, and cost-effectiveness. Figure 5a represents SWASV curves obtained for 0.5 µM each of Pb(II) and Cd(II) in 0.1 M sodium acetate buffer at different pH (3 to 5.5). It can be seen from the figure that when the pH of the solution was at 4.5 the stripping peak currents obtained for Cd(II) and Pb(II) ions were in the nearby range as compared to peak currents at other pH, which can also be depicted in Fig. 5b. Hence, to achieve maximum sensitivity for simultaneous determination of Cd(II) and Pb(II) ions, pH 4.5 was selected for further experiments. To attain maximum sensitivity, deposition time is another important parameter that is to be considered while performing stripping analysis. Therefore, to optimize the deposition time SWASV was performed with a deposition potential of − 1.1 V with 0.5 µM of Pb(II) and Cd(II) in 0.1 M sodium acetate buffer pH 4.5, with varying deposition time (40, 80, 120, 160, 200, 240, 280 s). As shown in Fig. 5c the stripping peak current increases with an increase in the deposition time, but there was no considerable increase in peak current obtained with increasing the deposition time beyond 240 s. Hence, considering the a﻿nalysis time and in order to achieve high sensitivity, a deposition time of 240 s was chosen.

The observed slight variations in peak potentials at different pH may be due to alteration of the charge on the electrode surface. The surface charge influences the adsorption of metal ions and can affect the kinetics of oxidation reactions, leading to shifts in oxidation potentials.

### Calibration curve

**Fig. 6:**
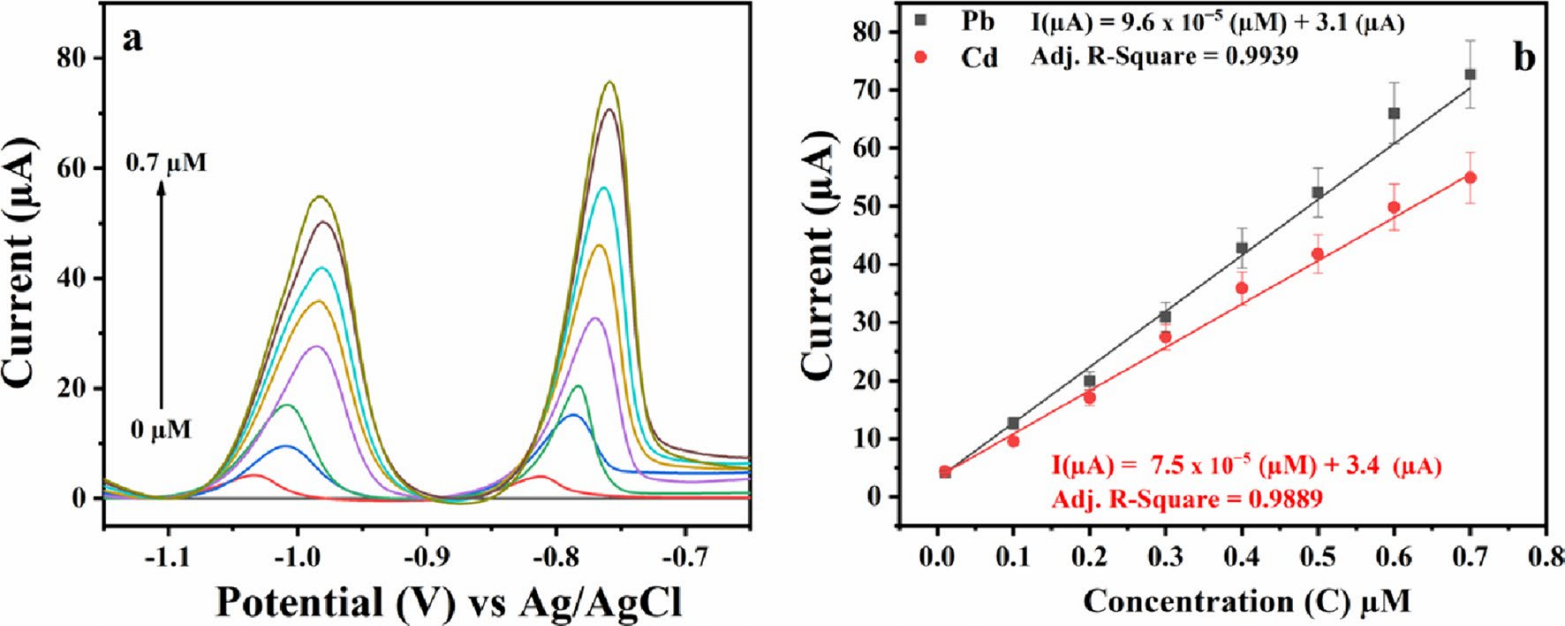
**a** SWASV for different concentrations of Pb(II) and Cd(II) ions in 0.1 M acetate buffer pH 4.5, **b** linear plots for the increasing concentration of Pb(II) and Cd(II) ions

The analytical performance of the Cyst/4-CP/SPCE for quantification of Cd(II), and Pb(II) was evaluated by SWASV under the optimized experimental parameters (Fig. 6a). It can be seen from the figure that with increasing metal ion concentration the stripping peak current increases. The sensor response was linear (Fig. 6b) in the concertation range of 0.01 µM to 0.7 µM. The sensor exhibited an excellent linear-concentration range and the linear-regression equations obtained for Pb(II) and Cd(II) are as follows

1 $${\text{I}}\left( {\mu {\text{A}}} \right) \, = { 9}.{\text{6 x 1}}0^{ - {5}} \left[ {{\text{Pb}}} \right] \, \left( {\mu {\text{M}}} \right) \, + { 3}.{1}\left[ {\mu {\text{A}}} \right]$$

2 $${\text{I}}\left( {\mu {\text{A}}} \right) \, = { 7}.{\text{5 x 1}}0^{ - {5}} \left[ {{\text{Cd}}} \right] \, \left( {\mu {\text{M}}} \right) \, + { 3}.{4}\left[ {\mu {\text{A}}} \right]$$

The LOD was calculated from 3S_b_/b where Sb is the standard deviation of blank measurement (*n* = 10) and b is the slope of linearity. The LOD was found to be 0.65 nM (0.09 PPB) for Pb(II) and 0.882 nM (0.134 ppb) for Cd(II).

We have compared our study with some other methods and previously reported electrochemical sensors for Pb(II) and Cd(II) and the data are summarized in Table 1.

**Table 1:** Comparison of previously reported electrochemical sensor for Pb(II) and Cd(II) with our sensor

Modified electrode	Technique used	Linear range Cd(II) (µg L^−1^)	Linear range Pb(II (µg L^−1^)	LOD Cd(II) (µg L^−1^)	LOD Pb(II) (µg L^−1^)	Sample	References
GSH-SPCE	SWASV	10–200	10–200	15	11	Water	[30]
ERGNO/Bi/SPE	SWASV	1—60	1—60	0.5	0.8	Milk	[35]
L-cys/GR-CS/GCE	DPASV	0.56–67.2	1.04–62.1	0.45	0.12	Honey/Rice	[36]
Engineered MWCNTs/GCE	SWASV	2–50	2–50	0.4	0.3	Water	[29]
AgNP/Bi/Nafion/CSPEs	SWASV	0.5–400	0.1–500	0.5	0.1	Water	[37]
Nafion/CLS/PGR/GCE	DPASV	5.60–560	10.36–1036	0.34	2.07	Water	[38]
N@LEG/GCE	SWASV	5–380	0.5–380	1.08	0.16	Water	[39]
g-C_3_N_4_/SPE	SWASV	30–120	30–110	21.8	10.4	Water	[40]
SWCNHs/SPE	SWASV	1–60	1–60	0.2	0.4	Milk/ Honey	[41]
p-PDMS@MSF/ITO	DPV	30–900	4–1500	2	4	Beverage	[42]
NH_2_-Ti_3_C_2_T_x_/SPE	DPASV	5–100/100–500	10–100/100–500	0.36	0.31	Food	[43]
Cyst/4-CP/SPCE	SWASV	1.12–80	2–45	0.092	0.134	Water	This work

*ERGNO* electrochemically reduced graphene oxide, *Bi* Bismuth, *SPE* screen-printed electrode, *L-cys* L-cysteine, *GR-CS* graphene-chitosan, *GCE* glassy carbon electrode, *MWCNTs* multi-walled carbon nanotubes, *AgNP* silver nanoparticles, *CSPEs* Carbon stencil-printed electrodes, *CLS* calcium lignosulphonate, *PGR* porous graphene, *g-C*_*3*_*N*_*4*_ graphitic carbon nitride, *SWCNHs* Single-walled carbon nanohorns, *PDMS* polydimethylsiloxane

### Reproducibility, and stability

Five electrodes modified in the same batch and under identical conditions were used to check the reproducibility of the modified electrode. SWASV was performed on all electrodes using 0.5 µM of Pb(II) and Cd(II) in 0.1 M sodium acetate buffer pH 4.5, at optimized experimental parameters. The stripping peak currents obtained at different sensors were in close agreement, and the relative standard deviation (RSD) was found to be 3.24%.

The storage stability of the modified electrodes was examined at room temperature for 28 days. Moreover, the modified sensors were stable and 94% of the original response was retained toward 0.5 µM of Pb(II) and Cd(II).

### Interference study

**Fig. 7:**
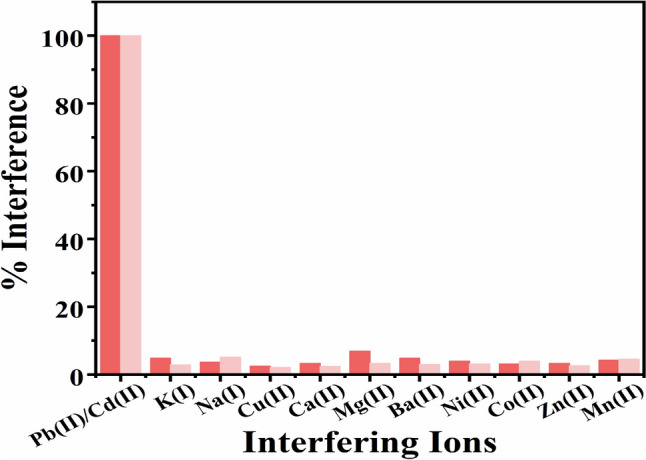
The influence of commonly found metal ions in water samples toward determination of Pb(II) and Cd(II) ions in 0.1 M acetate buffer pH 4.5

Various possible interfering ions were added to the solution of 0.5 µM Pb(II) and Cd(II) to validate the developed procedure for analyzing Pb(II) and Cd(II) in real samples and to demonstrate the selectivity of this new method. The experimental results reveal that 100 folds increase in the concentration of interfering ions [K(I), Na(I), Cu(II), Ca(II), Mg(II), Ba(II), Ni(II), Co(II), Zn(II), Mn(II)] did not affect the selectivity of the electrode at pH 4.5 toward Pb(II) and Cd(II). Figure 7 represents the percentage interference which is calculated from the difference between the electrochemical response measured in the presence and absence of the interfering ions to the response obtained in the absence of interfering ions. It is typically expressed as the percentage change in the measured peak currents caused by the interfering ions.

Mathematically, the percentage interference can be expressed as:

$${\text{Percentage }} {\text{interference }} \left( \% \right) = \frac{{{{\mid }}I_{Interference} - I_{Pure} {{\mid }}}}{{I_{Pure} }}{{ \times 100}}$$

I_Interference_: Peak current observed in the presence of interfering ions.

I_Pure_: Peak current observed in the absence of interfering ions.

### Real-sample analysis

The developed sensor Cyst/4-CP/SPCE was employed for the analysis of different water samples spiked with 50 ppb of Pb(II) and Cd(II) ions. The results obtained were correlated with one of the conventional methods (ICP-AES) used for metal ion analysis and summarized in Table 2. The obtained percentage recoveries of Pb(II) and Cd(II) ions on Cyst/4-CP/SPCE are in the range of 93.6% to 104.2% with a mean RSD of 2.68%.

**Table 2:** Recovery study for water sample and comparison with ICP-AES

Samples	Concentration added (ppb)	Concentration found (ppb)						ICP-AES	
		Cd(II)	% Recovery	% RSD	Pb(II)	% Recovery	% RSD	Cd(II)	Pb(II)
								(ppb)	
Drinking water	0	ND	−	−	ND	−	−	ND	ND
	20	19.7	98.5	1.02	19.4	97	0.95	18.6	19.2
	50	48.9	97.8	1.3	49.5	99	1.38	48	50
Tap water	0	ND	−	−	ND	−	−	ND	ND
	20	19.8	99	0.702	19.1	95.5	0.77	19.2	20.5
	50	50.6	101.2	1.25	50.5	101	1.55	51	50
Sewage water	0	ND	−	−	ND	−	−	ND	ND
	20	19.6	104.2	0.85	20.3	101.5	0.91	21	20.8
	50	50.8	101.6	1.36	49.9	99.8	1.71	54	52

*ND Not detected

## Conclusion

In this work, a novel electrochemical sensor based on chemically modified covalently bonded thiol functionalized screen-printed carbon electrode was developed. The modified sensor was utilized for the determination of Pb(II) and Cd(II) by SWASV. The modified electrode exhibited enhanced selectivity and sensitivity toward lead and cadmium ions, due to the high complexing ability of the thiol functional group present at the surface of the electrode. Selectivity, storage stability, and reproducibility of Cyst/4-CP/SPCE toward Pb(II) and Cd(II) were investigated. The proposed sensor was used in the determination of lead and cadmium ions in real water samples and the results were in satisfactory agreement with ICP-AES determination. The developed Cyst/4-CP modified sensor can be used as an excellent alternative to conventional spectroscopic techniques.

### Supplementary Information

Below is the link to the electronic supplementary material.

Supplementary file1 (DOCX 107 KB)
