# Supplementary material for: Thiol-based chemically modified carbon screen-printed electrode for simultaneous quantification of trace level Pb(II) and Cd(II)
Source: Anal Sci. 2024 May 14;40(8):1449–57. doi: 10.1007/s44211-024-00581-z (PMC11269355; doi:10.1007/s44211-024-00581-z)
Supplement: Supplementary file 1 — Supplementary file1 (DOCX 107 KB) [file 44211_2024_581_MOESM1_ESM.docx]

**Thiol-based chemically modified carbon screen-printed electrode for simultaneous quantification of trace level Pb(II) and Cd(II)**

Mritunjay S Tiwari, Arun K Kadu*^[a]^

University Department of Chemistry, University of Mumbai, Vidyanagari, Santacruz (East), Mumbai-400 098, India ^[a]^

*Corresponding Author mob.: +91 9764562013

Email: [kadu.arun@chemistry.mu.ac.in](mailto:kadu.arun@chemistry.mu.ac.in) (Arun K Kadu)

**Cysteamine Immobilization on 4-CP/SPCE**

**Fig S1 (a)**, represents SWASV plots for 0.5 µm of Cd(II) and Pb(II) ions in acetate buffer pH 4.5, at a deposition potential of −1.1 V with a preconcentration time of 120 s, at varying incubation time for immobilization of cysteamine on 4-CP/SPCE. It can be seen from the figure that as the time of incubation increases from 1 hour to 2 hours, there is an increase in peak current, and also a shift in peak potential was observed which indicates immobilization of cysteamine on the surface of 4-CP/SPCE. Increasing the incubation time after 3 hours does not add any significant change in the peak currents **Fig S1 (b)**, which may indicate complete immobilization of cysteamine on 4-CP modified SPCE.

|  |  |
| --- | --- |
| S1(a)SWASV (b) Time vs peak current at Cyst/4-CP/SPCE plot with increasing immobilization time of cysteamine for 0.5 µm of Pb(II) and Cd(II) in 0.1 m sodium acetate buffer pH 4.5. | |
